# Multidrug-Resistant Bacterial Infections in Pediatric Patients Hospitalized at King Abdulaziz University Hospital, Jeddah, Western Saudi Arabia

**DOI:** 10.3390/children11040444

**Published:** 2024-04-07

**Authors:** Fajr A. Saeedi, Moustafa A. Hegazi, Hani Alsaedi, Ahmed Hussain Alganmi, Jawahir A. Mokhtar, Eilaf Majdi Metwalli, Hanaa Hamadallah, Ghassan S. Siam, Abdullah Alaqla, Abdullah Alsharabi, Sultan Ahmed Alotaibi

**Affiliations:** 1 Department of Pediatrics, Faculty of Medicine in Rabigh, King Abdulaziz University, Jeddah 80205, Saudi Arabia; fasaeedi@kau.edu.sa (F.A.S.); hasalsaede@kau.edu.sa (H.A.); hhamdallah@kau.edu.sa (H.H.); 2 Department of Pediatrics, Faculty of Medicine in Mansoura, Mansoura University Children’s Hospital, Mansoura 35516, Egypt; 3 Faculty of Medicine in Rabigh, King Abdulaziz University, Jeddah 80205, Saudi Arabia; dr.ahmedalganmi@gmail.com (A.H.A.); abdullahalegla@gmail.com (A.A.); abdullahalsharabi1899@gmail.com (A.A.); sultanahmedx1@gmail.com (S.A.A.); 4 Department of Clinical Microbiology and Immunology, Faculty of Medicine, King Abdulaziz University Hospital, Jeddah 80215, Saudi Arabia; jmokhtar@kau.edu.sa; 5 Vaccines and Immunotherapy Unit, King Fahad Medical Research Center, King Abdulaziz University, Jeddah 80216, Saudi Arabia; 6 Faculty of Medicine, King Abdulaziz University, Jeddah 80215, Saudi Arabia; eilaf.metwalli@gmail.com

**Keywords:** multidrug-resistant bacteria, hospitalized pediatric patients, Jeddah, Saudi Arabia

## Abstract

Multidrug-resistant bacterial infections (MDRIs) constitute a major global threat due to increased patient morbidity/mortality and hospital stay/healthcare costs. A few studies from KSA, including our locality, addressed antimicrobial resistance in pediatric patients. This study was performed to recognize the incidence and clinical/microbiologic features of MDRIs in hospitalized pediatric patients. A retrospective cross-sectional study included pediatric patients < 18 years, admitted to King Abdulaziz University Hospital, between October 2021 and November 2022, with confirmed positive cultures of bacteria isolated from blood/body fluids. Patients’ medical files provided the required data. MDR organisms (MDROs) were identified in 12.8% of the total cultures. The incidence of MDRIs was relatively high, as it was detected in 42% of patients and in 54.3% of positive bacterial cultures especially among critically ill patients admitted to the NICU and PICU. Pneumonia/ventilator-associated pneumonia was the main type of infection in 37.8% of patients with MDROs. *Klebsiella pneumoniae* was the most common significantly isolated MDRO in 39.5% of MDR cultures. Interestingly, a low weight for (no need for their as terminology weight for age is standard and well-known) was the only significant risk factor associated with MDROs (*p* = 0.02). Mortality was significantly higher (*p* = 0.001) in patients with MDROs (32.4%) than in patients without MDROs (3.9%). Patients who died including 85.7% of patients with MDROs had significantly longer durations of admission, more cultures, and utilized a larger number of antibiotics than the surviving patients (*p* = 0.02, *p* = 0.01, *p* = 0.04, respectively). This study provided a comprehensive update on the seriously alarming problem of MDROs, and its impacts on pediatric patients. The detected findings are crucial and are a helpful guide to decid for implementing effective strategies to mitigate MDROs.

## 1. Introduction

Antibiotics are one of the greatest discoveries of the previous century. Antibiotics have widespread global usage among pediatric patients to essentially treat various bacterial infections [1,2]. Antibiotics are considered double-edged weapons because they possess the capacity to either eliminate or impede the proliferation of bacteria if they are used appropriately, but they may also have side effects, including antimicrobial resistance (AMR). The problem of AMR arises when bacteria escape the stress of antibiotic exposure through various mechanisms such as mutational changes, acquiring genetic material, or modifying gene expression. This results in bacterial adaptation and the emergence of new strains that are resistant to the available antibiotics [3,4].

The indiscriminate use of antibiotics by clinicians, or inappropriate utilization by an individual, can have serious effects on other individuals related to the appearance of multidrug-resistant (MDR) bacteria. This phenomenon arises when a bacterium carries multiple resistance genes simultaneously [3,4].

Okomo et al. (2019) showed that a significant proportion of neonatal invasive bacterial infections in the sub-Saharan African region are mainly attributed to antibiotic-resistant *Staphylococcus aureus*, *Klebsiella pneumoniae*, and *Escherichia coli* [5].

Moreover, even in developed countries, Fleming et al. found that approximately 30% of administered antibiotics in the United States are considered inappropriate [6]. It has been estimated that antibiotic-resistant microorganisms have led to 2.8 million infections and 35,000 fatalities annually in the United States [7]. According to McCullough et al. (2017), antibiotic prescriptions for acute respiratory infections in Australia exceed the clinical guideline recommendations by four–nine times [8].

The rapidly spreading global pandemic of MDR organisms, characterized by acquired resistance to at least one agent in three or more clinically utilized antimicrobial classes, poses a substantial threat [9]. This phenomenon represents a significant medical and global public health crisis, leading to increased patient morbidity, mortality, prolonged hospital stays, and escalated healthcare costs [10,11].

The substantial increase in serious global health concern regarding the terror of multidrug-resistant organisms (MDROs) or superbugs is related to their adverse effects on economic, social, and environmental targets of the sustainable development goals [12]. Predictions indicate that deaths from MDR superbugs could surge from 700,000 to 10 million annually, with estimated costs reaching as high as US $100 trillion worldwide by 2050. Alarmingly, anticipated fatalities from MDR infections are expected to surpass even those from cancer [13].

The causes of MDR can be attributed to various factors, including the irrational overuse or abuse of antimicrobials, incorrect dosage, and repetitive use of a particular drug in humans, veterinary medicine, and agriculture coupled with too-few antibiotics in the pipeline to tackle the global crisis of MDR. Over the past three decades, the rate of new-antibiotic development and approval has steadily declined, with only four new antibiotics approved in 2014 leading to limited options for treating resistant bacteria [3,14,15].

Health care-associated infections (HAIs) with MDROs are notably prevalent among hospitalized patients, particularly those in intensive care units (ICUs), who face high risks of recurrent infections, elevated morbidity and mortality rates, and increased treatment expenses due to frequent failures of empirical antimicrobial therapy and prolonged hospitalization [16,17].

In Saudi Arabia, several challenges exist that could foster the emergence and dissemination of antimicrobial-resistant bacteria. Antibiotics are in the third rank among prescribed medications [18]. In light of the escalating reports on bacterial resistance in Saudi Arabia, there is a pressing need to investigate associations contributing to the escalation of antibiotic resistance [19].

According to nationwide surveillance of Gram-positive cocci in Saudi Arabia, approximately 32% of *Staphylococcus aureus* strains are methicillin-resistant *Staphylococcus aureus* (MRSA) [20]. Over the past decade, there has been a significant rise in the prevalence of carbapenem-resistant Gram-negative bacilli in Saudi Arabia compared to rates in the 1990s. This increase is accompanied by a growing prevalence of extended-spectrum beta-lactamase (ESBL) producing isolates, with some institutions reporting rates of 29% among *Escherichia coli* and 65% among *Klebsiella pneumoniae*. These escalating rates have been linked to numerous reported outbreaks, with associated mortality ranging between 11 and 40% [21,22].

In Saudi Arabia, few retrospective studies [20,21,23,24] have addressed antimicrobial resistance in general but not MDROs in particular in different areas of the kingdom (it is kept as kingdom because it refers to Saudi Arabia which is a kingdom). A relatively recent study identified a high rate of MDR *Acinetobacter baumannii* bacteremia in the southern region of Saudi Arabia, with approximately 50% of isolates classified as extensively drug-resistant isolates. These isolates were found to be sensitive to colistin but resistant to all other commonly used treatment options [25].

Regarding our locality, the problem of AMR bacteria especially MDROs has seldom been comprehensively addressed in Jeddah, in the western province of the kingdom as only a few studies have investigated the prevalence of AMR bacteria. These studies did not display the correlation of microbiological findings with clinical data of patients who had AMR bacteria [26,27].

The magnitude, risk factors, and pattern of the MDR problem vary significantly among different populations and regions within the country. Moreover, the susceptibility patterns of pathogens can differ greatly between hospitals and even within different units within the same hospital [27]. Therefore, this study aimed to investigate the incidence, clinical and microbiologic characteristics, risk factors, and impact of MDROs on pediatric patients admitted to King Abdulaziz University Hospital (KAUH) in Jeddah, western Saudi Arabia.

## 2. Subjects and Methods

### 2.1. Study Design and Setting

This retrospective cross-sectional study was conducted at a tertiary-level university hospital. The study included pediatric patients under the age of 18 who were hospitalized at KAUH between October 2021 and November 2022 and had confirmed positive cultures of bacteria isolated from blood or body fluids. Hospitalized patients from any admission location, whether from the inpatient pediatric ward, pediatric intensive care unit (PICU), or neonatal intensive care unit (NICU), were included. The study received approval from the research ethics committee of KAUH on 16 May 2023 (reference number 278-23). However, obtaining written informed consent from patients, parents, or caregivers was not required.

### 2.2. Data Collection

Data were retrieved from patient medical records available at the hospital information system. A structured checklist was utilized to gather all necessary information, including demographic characteristics, clinical manifestations, comorbidities, diagnosed type of infection, laboratory findings of culture and sensitivity results, admission location (pediatric ward, PICU, or NICU), administered treatment (number and class of administered antimicrobial agents, need for mechanical ventilation/tracheal intubation, use of corticosteroids, parenteral nutrition, and blood products), complications, length of stay, and outcome (survival to discharge, readmission within 30 days and death). Data of indwelling catheters including central vascular lines, urinary catheters, and endotracheal tubes (ETT) or tracheostomies were also recorded.

### 2.3. Exclusion Criteria

Patients with isolated or combined fungal infections, mycobacterial tuberculosis, and protozoal infections were excluded to avoid the potential contributing effects of non-bacterial infections on the interpretation of data. This exclusion aimed to focus the analysis specifically on cases of isolated MDR bacterial infections. Additionally, culture-positive patients with incomplete data in their medical records were also excluded from the study.

### 2.4. Definition of MDROs

MDROs are those who exhibit acquired resistance to at least one agent in three or more antimicrobial classes [9].

### 2.5. Statistical Analysis

The data were analyzed using Statistical Package for Social Sciences (SPSS) software version 25 (IBM Corporation, Armonk, NY, USA). Nominal or categorical data were presented as numbers and percentages, while numeric data were presented as means, ranges, medians, and interquartile range (IQR). Chi-square (χ^2^) and Fisher exact tests were utilized to compare nominal variables, while the Mann–Whitney U test was employed to compare medians. A *p* value < 0.05 was considered as statistically significant.

## 3. Results

This study included 88 patients; 23 neonates, 27 infants, 30 children, and 8 adolescents with a median age of 11.5 month, IQR of 1.25–46.25 month, and range from 1 day to 13 years. Fifty-five patients (62.5%) were males, while 33 patients (37.5%) were females. Saudi and non-Saudi patients represented 43.2% and 56.8% of patients, respectively. The PICU and NICU were the admission places for 54.5% of patients, while 45.5% of patients were admitted into pediatric wards. Pneumonia/ventilator-associated pneumonia was the main type of diagnosed infection in 40.9% of patients followed by blood stream infection including early- and late-onset neonatal sepsis and catheter-associated blood stream infection (CABSI) in 20 out of 88 patients (22.8%). Mutidrug (Multidrug can be one word as in title)resistant infections were diagnosed in 37 patients (42.0%). Forty-two patients (47.7%) were considered to have nosocomial, healthcare, or hospital-acquired infections (HCAIs), which were typically absent at the time of admission but developed after hospitalization and manifested 48 h after admission. The majority of patients, 66/88 (75%), had different comorbidities, 36/88 patients (40.9%) required mechanical ventilation, 27/88 patients (30.7%) required central venous catheter, and 48/88 patients (54.5%) required treatment with corticosteroids or blood products. Regarding the outcomes of patients, 47/88 (53.4%) survived to discharge, and 14/88 (15.9%) died. The demographic and clinical characteristics of 88 pediatric patients with positive cultures of bacterial isolates admitted at KAUH between October 2021 and November 2022 are presented in Table 1.

Forty-nine patients had a single culture, while 39 patients had multiple cultures. A total of 1749 cultures were performed for pediatric patients hospitalized at KAUH during the same period of the study. Four-hundred and forty-one cultures were positive for bacterial isolates. Multidrug-resistant bacteria represented 223/1749 (12.8%) of the total cultures and 223/411 (54.3%) of the positive cultures. The most commonly isolated organisms were *Klebsiella pneumoniae* in 122 cultures (29.7%), *coagulase negative staphylococci* (CONS) mainly *Staphylococcus epidermidis* in 67 cultures (16.3%), and *MRSA* in 61 cultures (14.8%). The main two sites of isolated organisms were the blood stream and CABSI in 42.8% of patients followed by the respiratory tract and ETT in 30.7% of patients. For *Klebsiella pneumoniae*, 62/122 (50.8%) were ESBL, 57/122 (46.7%) were Carbapenem resistant (CRE), and 3/122 (2.5%) were neither CRE nor ESBL. Extensively drug-resistant (XDR) *Klebsiella pneumoniae* was isolated from the respiratory tract and blood in 45 out of 122 cultures (36.9%), mainly from four male patients (three neonates with congenital heart disease and one 4-year-old child with heart disease and diabetes mellitus). These four patients had long hospital stays ranging from 67 to 127 days, one neonate survived to discharge, one neonate died, and the other two patients were readmitted to hospital within 30 days. For *Enterococcus faecium*, 3/28 (10.7%) were Vancomycin-resistant enterococci (VRE), and 25/28 (89.3%) were not VRE. For *Escherichia coli*, 17/26 (65.4%) were ESBL, 3/26 (11.5%) were CRE, and 6/26 (23.1%) were neither CRE nor ESBL. Regarding the sites of isolation of the most commonly detected organisms, *Klebsiella pneumoniae*: 52/122 (42.6%) were from blood, 36/122 (29.5%) were from the respiratory sample, and 23/122 (18.9%) were from urine. *For Coagulase negative staphylococci, mainly Staphylococcus epidermidis*, 52/67 (77.6%) were from blood and 7/67 (10.4%) were from the respiratory sample, and for *MRSA*, 24/61 (39.3%) were from blood, 18/61 (29.5%) were from the respiratory sample, and 13/61 (21.3%) were from soft tissues. Culture and sensitivity data of the 411 cultures are presented in Table 2.

A comparison between patients with and without MDRI is presented in Table 3. Patients with MDRI had significantly higher prevalence rates of low weight for age (75.7% vs. 47.1%, *p* = 0.02) and death (12/37 (32.4%) vs. 2/51 (3.9%), *p* = 0.001). No significant differences were detected between patients with and without MDRI regarding other studied variables.

A comparison between 223 cultures with MDROs and 188 cultures without MDROs revealed a significant difference regarding the isolated organisms (*p* < 0.0001). *Klebsiella pneumoniae* was isolated from 39.5% of the MDR cultures compared to 18.1% of the non-MDR cultures. On the other hand, CONS was isolated from only 11.7% MDRI cultures compared to 21.8% of the non-MDRI cultures. *Acinetobacter baumannii* was isolated from 3.6% of MDRI cultures, while it was not isolated from any non-MDRI culture. No significant difference was observed between both groups regarding the sites of isolated organisms. Table 4 shows the detailed comparison between MDR and non-MDR cultures.

No significant difference was found between the median age and IQR for patients with and without MDRI (7.5 (0.84–29) months versus 17 (2.0–60) months, and *p* = 0.08). Additionally, there was no significant difference between patients with and without MDRI regarding the duration of hospital admission (*p* = 0.79), number of cultures (*p* = 0.99), or number of antibiotics used in treatment (*p* = 0.88).

Regarding the outcomes, patients who died had significantly younger age and longer duration of admission than patients who were readmitted to hospital within 30 days (*p* = 0.02 and *p* = 0.03), respectively. Also, patients who died had significantly longer durations of admission, more cultures, and utilized greater numbers of antibiotics than the surviving patients (*p* = 0.02, *p* = 0.01 and *p* = 0.04), respectively. A comparison between deceased patients and patients who survived or were readmitted to hospital within 30 days is presented in Table 5.

## 4. Discussion

Antibiotic resistance is a global phenomenon, affecting healthcare systems worldwide. The rise in resistance rates varies among different regions and pathogens. “Superbugs” or MDRO are of paramount importance, as they pose a major threat to global health by challenging modern medicine as humans enter the post-antibiotic era [28].

Infectious diseases are responsible for approximately 60% of deaths among children, mostly because of their immature immune system. The use of antibiotics in children is also considered a double-edged sword. Studies have indicated a high susceptibility of children to MDRO infections, with Gram-negative bacterial infections constituting a significant portion at 67.98%. Among these, ESBL-producing *Escherichia coli* and *Klebsiella pneumoniae* are frequently identified. Moreover, *MRSA* poses a significant threat as a potential serious bacterial infection in children [29].

In Saudi Arabia, studies have documented escalating rates of antibiotic resistance and MDRI [19,21,22,23,24,25,30,31,32]. There is a need for periodical evaluation of the magnitude and risk factors of MDROs to adapt an appropriate prevention and control strategy. Therefore, this study was conducted to have an update on the incidence, clinical and microbiologic features, risk factors, and impact of MDROs on pediatric patients admitted at KAUH.

In this study, 411 cultures were positive for bacterial isolates in 88 pediatric patients hospitalized at KAUH between October 2021 and November 2022 as 39/88 patients have multiple cultures. The patients included 23 neonates, 27 infants, 30 children, and 8 adolescents with a median age of 11.5 month, and an IQR of 1.25–46.25. MDROs represented only 223/1749 (12.8%) of the total cultures performed for hospitalized pediatric patients during the same period of the study. However, MDROs were identified in a significant proportion of patients 37/88 (42%) and in more than half of cultures 223/411 (54.3%), representing the alarming serious problem of MDRI and its adverse consequences in pediatric patients hospitalized at KAUH. Nosocomial or HCAI was considered in 42/88 of total patients (47.7%) and in 16/37 (43.2%) of patients with MDROs. At KAUH, standard precautions for infection prevention and control are mainly adopted from CDC and the Healthcare Infection Control Practices Advisory Committee, and an antibiotic stewardship program, including infectious disease consultation, is implemented with a moderate level of compliance with the standard precautions [33]. Similar trends have been reported for MDROs in China, where the prevalence of *MRSA* stands at approximately 35%, the prevalence of ESBL-producing *Escherichia coli* is still higher than 55%, the prevalence of carbapenem-resistant *Pseudomonas aeruginosa* persisted at 20% over time, and the prevalence of carbapenem-resistant *Acinetobacter baumannii* has constantly increased to 60% [34].

However, the prevalence of MDROs is lower than the reported MDR Gram-negative bacteria of 89.5% in Ghana [35] and MDR hospital-associated infections of 52% in PICU of a university hospital in Thailand [36], but it is (it is used because it refers to prevalence) higher than the reported MDROs of 9.2% in Egypt [37]. It is clearly evident that there is marked variation in the prevalence of MDROs which can be attributed to differences in demographic characteristics, sample size, place of admission within the hospital, and implementation of infection control measures. However, whatever the rate of MDROs, it seems that MDROs and their consequences are a major threat and serious concern particularly that MDROs’ prevalence is expected to continually rise globally.

In this study, the median and IQR for patients with MDRI was 7.5 and (0.84–29) months, 24/37 (64.9%) were males, 36/37 (97.3%) patients were neonates, infants, or children, and more than half of patients 21/37 (56.7%) were admitted either in the PICU or NICU. Pneumonia/ventilator-associated pneumonia was the main type of diagnosed infection with isolated MDROs from respiratory samples in 14/37 patients (37.8%) followed by the same rate of isolated MDROs in 8/37 patients (21.6%) in both blood streams including in early and late onset and CABSI and urine. Similarly, these findings were recorded in a relatively recent study from Egypt, as 61.5% of patients with MDRI in PICU had ages of less than 1 year, but only 50% of patients were males, and sputum samples were the most common site of isolated MDROs (59.3%) followed by blood (31.2%) and urine (9.4%) [37].

In this study, insignificant differences were detected in the demographic and for other risk factors between patients with and without MDROs (Table 3), except for low weight for age which was significantly lower in patients with MDROs compared to patients without MDROs (*p* = 0.02, Table 3). This can be explained by the special characteristics of the included patients, as more than half of the total patients 48/88 (54.5%) were critically ill patients admitted in either the NICU or PICU including closely similar proportions of patients with MDROs, 21/37 (56.8%), and patients without MDROs, 27/51 (52.9%) (Table 3). Moreover, these two groups of critically-ill patients with or without MDROs demonstrated statistically insignificant differences in known risk factors for MDROs, such as initial underlying comorbidities, previous antibiotic use, and need for mechanical ventilation and central vascular line insertion.

Interestingly, low weight for age was the only significant and determinant risk factor associated with MDROs. Low weight for age is an evident indicator and closely correlated with undernutrition. Undernutrition in children is strongly associated with elevated morbidity and mortality rates, primarily due to the increased incidence of severe infections. Undernutrition is a widely recognized critical factor and a common cause of secondary immune deficiency, particularly affecting T-cell-mediated immune responses. Undernourished children exhibit significantly reduced expression levels of key cytokines involved in T-cell function, including IFN-γ, IL-2, IL-12, IL-18, and IL-21 (Th1 cytokines), compared to well-nourished children [38,39].

Consistent with the findings of this study, another investigation specifically highlighted that bacteremia, particularly when caused by MDR bacteria, is associated with increased morbidity and mortality rates in malnourished children under 5 years of age [40].

A recent study revealed a high incidence of intestinal carriage and invasive infection with intestinal-derived MDROs, including ESBL- and carbapenemase-producing Enterobacterales, in malnourished children. The risk of infection with intestinal-derived organisms is heightened in cases of undernutrition due to impairments in intestinal mucosal barrier function and innate and adaptive immunity. The interplay between intestinal microbiota and diet influences nutritional status with significant impact on infectious outcomes [41]. A vicious cycle between nutrition and infection is elicited, where undernutrition compromises barrier function, facilitating easier access by pathogens, and impairing immune function, reducing the host’s ability to eliminate pathogens, predisposing them to infections. On the other hand, infections can alter nutritional status by affecting nutrient requirements, dietary intake, and absorption. Thus, the presence of infections can exacerbate the severity of the undernourished state which leads to persistent or recurrent infections, and the cycle continues. The global burden of undernutrition and infectious diseases is substantial, especially among children with a particularly increased risk of antimicrobial resistance mostly related to increased exposure to HCAIs and antimicrobials [42]. Consequently, in this study, it can be expected that not only low weight for age or undernutrition was a significant risk factor of MODROs, but MDROs could exacerbate the severity of undernutrition leading to more severe persistent or recurrent MDRI, resulting in adverse outcome especially among patients who had a prolonged hospital stay and fatal outcome.

In this study, the most important detected significant difference was related to the outcome of patients as the mortality rate was statistically significantly higher (*p* = 0.001) in patients with MDROs (32.4%) than in patients without MDROs (only 3.9%), Table 3. A closely similar mortality rate was reported in another study, as 34.6% of patients with MDROs died [36]. The fatal outcome and several problems associated with MDROs as well as their expected future serious consequences have been demonstrated in multiple studies [10,11,13]. Similarly, in this study, patients who died including 12/14 patients (85.7%) with MDROs had significantly longer durations of admission, more cultures, and utilized a greater number of antibiotics than surviving patients (*p* = 0.02, *p* = 0.01 and *p* = 0.04), respectively (Table 5). However, it is difficult to conclude whether the longer hospital stays associated with MDROs is the cause/risk factor or the effect of infection with MDROs.

Although most well-known risk factors for MDROs except low weight for age were not significantly different between patients with and without MDROs, MDROs were associated with a significantly fatal outcome which is an extremely alarming finding necessitating the proper management of MDROs with high levels of specialized healthcare resources, including laboratory diagnostics, antimicrobial stewardship programs, proper surveillance, and implementation of infection control measures in view of the currently limited antibiotic options to treat MDR bacteria.

Regarding culture and sensitivity data, significant differences in isolated MDROs were found (*p* = 0.001, Table 4). *Klebsiella pneumoniae* was significantly more isolated from 39.5% of the MDR cultures compared to 18.1% of the non-MDR cultures. *Acinetobacter baumannii* was significantly more isolated from 3.6% MDR cultures, but it was not isolated from any non-MDR culture. CONS was significantly more isolated from 21.8% of non-MDRI cultures compared to only 11.7% of MDRI. Rezk et al. [37] reported that the most prevalent MDR isolated organisms in PICU were *Acinetobacter baumannii*, *Klebsiella pneumoniae*, and *Pseudomonas* with 50%, 42.8%, and 7.1%, respectively. Wang et al. [43] reported that the most common isolated resistant organisms were *Acinetobacter baumannii*, *MRSA, and Pseudomonas* in 35.4%, 30.4%, and 27.8% of PICU cases, respectively.

Despite the fact that this is a single-center study, it is worth mentioning its strength points. Firstly, it is one of few comprehensive studies conducted in our locality to provide a recent update on the status of the seriously alarming problem of MDROs in hospitalized pediatric patients. Secondly, it provided updated information on the incidence of MDROs with a correlation of patients’ demographic, clinical, risk factors, and impact of MDROs to microbiologic culture and sensitivity data in a considerable number of patients, including the different pediatric age groups (from neonates to adolescents) admitted at different places within the hospital (inpatient wards, NICU, and PICU). Finally, this study contributes valuable insights from Saudi Arabia, an area outside the geographical scope of previous relevant studies, which have primarily focused on regions such as the USA, Europe, and China.

However, the limitations of this study should be mentioned. Firstly, it is a single-center retrospective study, which may limit the generalizability of the findings to other settings. Secondly, there is a lack of long-term follow-up of the studied group, which restricts the ability to assess outcomes over time. Additionally, it is challenging to determine whether certain variables, such as low weight for age and prolonged hospital stay, which were significantly associated with MDROs, are causes/risk factors or effects of infection with MDROs in hospitalized pediatric patients.

## 5. Conclusions

This study revealed that the presence of MDROs is a significant HCAI problem and a major threat, especially among critically ill pediatric patients admitted at the NICU and PICU of KAUH, Jeddah, western Saudi Arabia. The incidence of MDROs between October 2020 and November 2021 was relatively high, as MDROs were identified in a significant proportion of patients 37/88 (42%) and in more than half of cultures 223/411 (54.3%) representing the alarming serious problem of MDROs and its adverse consequences in hospitalized pediatric patients. Pneumonia/ventilator-associated pneumonia was the main type of diagnosed infection with isolated MDROs from respiratory samples in 14/37 patients (37.8%). *Klebsiella pneumonia* was the most common significantly isolated MDRO in 39.5% of the MDR cultures. Extensively drug-resistant *Klebsiella pneumoniae* was isolated from the respiratory tract and blood in 45 out of 122 cultures (36.9%) mainly from four critically ill male patients. Interestingly, low weight for age emerged as the sole significant and determining risk factor associated with MDROs, which was significantly lower in patients with MDROs compared to patients without MDROs (*p* = 0.02). MDROs had a serious impact on hospitalized pediatric patients as the mortality rate was statistically significantly higher (*p* = 0.001) in patients with MDROs (32.4%) than in patients without MDROs (only 3.9%), and patients who died including 12/14 patients (85.7%) with MDROs had significantly longer admission durations, more cultures, and utilized a larger number of antibiotics than surviving patients (*p* = 0.02, *p* = 0.01, and *p* = 0.04).

## Figures and Tables

**Table 1:** Demographic and clinical characteristics of 88 pediatric patients with positive cultures of bacterial isolates admitted at KAUH between October 2021 and November 2022.

Variables	Number	%
**Age category (Median age 11.5, IQR: 1.25–46.25 mo, Range: 1 day–13 years)**		
Neonate	23	26.1
Infant	27	30.7
Child	30	34.1
Adolescent	8	9.1
**Gender**		
Males	55	62.5
Females	33	37.5
**Residence**		
Jeddah	82	93.2
Makkah	5	5.7
Taif	1	1.1
**Nationality**		
Saudi	38	43.2
Non-Saudi	50	56.8
**Place of admission**		
In-patient ward	40	45.5
PICU	29	32.9
NICU	19	21.6
**Weight for age**		
Low weight for age	52	59.1
Normal weight for age	32	36.4
High weight for age	4	4.5
**Mutidrug-resistant infections**	37	42.0
**Nosocomial or healthcare-associated infection**	42	47.7
**Diagnosed type of infection**		
Pneumonia/ventilator-associated pneumonia	36	40.9
Multiple-site infection (pneumonia and meningitis)	4	4.5
Early-onset neonatal sepsis	8	9.1
Late-onset neonatal sepsis	5	5.7
Skin and soft tissue infection	7	8.0
Central nervous system infections	5	5.7
Urinary tract infections	15	17.0
Gastrointestinal infections (peritonitis)	1	1.1
Catheter-associated blood stream infection	7	8.0
**Comorbidities**		
None	22	25.0
Cardiovascular disease	29	33.0
Neurologic disease	8	9.1
Malignancy	11	12.5
Renal disease	9	10.2
Pulmonary disease	4	4.5
Diabetes mellitus	1	1.1
Hematologic disease (SCA)	1	1.1
Liver disease	3	3.4
**Previous antibiotic use in the preceding 3 months**	13	14.8
**Mechanical ventilation**	36	40.9
**Central vascular line**	27	30.7
**Need for treatment with steroids**	48	54.5
**Need for TPN**	23	26.1
**Need for blood products**	48	54.5
**Outcome**		
Survival to discharge	47	53.4
Readmission within 30 days	27	30.7
Death	14	15.9

KAUH: King Abdulaziz University Hospital, IQR: interquartile range, SCA: sickle cell anemia, TPN: total parenteral nutrition.

**Table 2:** Culture and sensitivity data of 411 cultures for 88 pediatric patients admitted at KAUH between October 2021 and November 2022.

Variables	Number	%
**Number of cultures/patient (Median: 2, IQR: 1–4)**		
Single culture/patient	49	55.7
Multiple cultures/patient	39	44.3
**Multidrug-resistant cultures**	223	54.3
**Isolated organisms**		
*Klebsiella pneumoniae*	122	29.7
*Coagulase negative staphylococci mainly S. epidermidis*	67	16.3
*Methicillin resistant staphylococcus aureus*	61	14.8
*Enterobacter coloaca*	8	1.9
*Escherichia coli*	26	6.3
*Enterococcus gallinarum*	2	0.5
*Diphtheroids*	5	1.2
*Enterococcus faecium*	28	6.8
*Streptococcus mitis*	3	0.7
*Salmonella*	3	0.7
*Serratia marcescens*	11	2.7
*Pseudomonas aeruginosa*	36	8.8
*Staphylococcus aureus*	16	3.9
*Streptococcus viridans*	2	0.5
*Acinetobacter baumannii*	8	1.9
*Stenotrophomonas maltophilia*	10	2.4
*Chryseobacterium meningosepticum*	1	0.2
*Enterobacter aerogenes*	2	0.5
**Sites of isolated organisms**		
Respiratory tract and ETT	126	30.7
Blood stream and CABSI	176	42.8
Skin and soft tissue	40	9.7
Urine/urinary catheter	59	14.4
Peritoneal fluid	6	1.5
CSF	4	1.0

KAUH: King Abdulaziz University Hospital, IQR: interquartile range, ETT: endotracheal tube, CABSI: catheter-associated blood stream infection, CSF: cerebrospinal fluid.

**Table 3:** Comparison between pediatric patients with and without isolated multidrug-resistant infectious organisms (MDROs) admitted at KAUH between October 2021 and November 2022.

Variables	Patients with MDRI (N = 37)		Patients without MDRI (N = 51)		Test *	
	Number	%	Number	%	X^2^	*p*
**Age category (Median age 11.5, IQR: 1.25–46.25 mo, Range: 1 day–13 years)**					4.6	0.20
Neonate	12	32.4	11	21.6		
Infant	13	35.1	14	27.5		
Child	11	29.7	19	37.3		
Adolescent	1	2.7	7	13.7		
**Gender**					0.15	0.69
Males	24	64.9	31	60.8		
Females	13	35.1	20	39.2		
**Residence**					2.15	0.34
Jeddah	33	89.2	49	96.1		
Makkah	3	8.1	2	3.9		
Taif	1	2.7	0	0.0		
**Nationality**					0.19	0.66
Saudi	17	45.9	21	41.2		
Non-Saudi	20	54.1	30	58.8		
**Place of admission**					0.29	0.86
In-patient ward	16	43.2	24	47.1		
PICU	12	32.4	17	33.3		
NICU	9	24.3	10	19.6		
**Weight for age**					8.4	0.02
Low weight for age	28	75.7	24	47.1		
Normal weight for age	9	24.3	23	45.1		
High weight for age	0	0.0	4	7.8		
**Nosocomial or healthcare-associated infection**	16	43.2	26	51.0	0.52	0.47
**Diagnosed type of infection**					11.8	0.23
Pneumonia/ventilator-associated pneumonia	14	37.8	22	43.1		
Multiple-site infection (pneumonia and meningitis)	2	5.4	2	3.9		
Early-onset neonatal sepsis	4	10.8	4	7.8		
Late-onset neonatal sepsis	3	8.1	2	3.9		
Skin and soft tissue infection	2	5.4	5	9.8		
Central nervous system infections	3	8.1	2	3.9		
Urinary tract infections	8	21.6	7	13.7		
Gastrointestinal infections (peritonitis)	0	0.0	1	2.0		
CABSI	1	2.7	6	11.8		
**Comorbidities**					4.4	0.82
None	9	24.3	13	25.5		
Cardiovascular disease	15	40.5	14	27.5		
Neurologic disease	4	10.8	4	7.8		
Malignancy	3	8.1	8	15.7		
Renal disease	4	10.8	5	9.8		
Pulmonary disease	1	2.7	3	5.9		
SLE under systemic steroids with DM	0	0.0	1	2.0		
Hematologic disease (SCA)	0	0.0	1	2.0		
Liver disease	1	2.7	2	3.9		
**Previous antibiotic use in the preceding 3 months**	6	16.2	7	13.7	0.11	0.75
**Mechanical ventilation**	17	45.9	19	37.3	0.67	0.41
**Central vascular line**	10	27.0	17	33.3	0.40	0.53
**Need for treatment with steroids**	22	59.5	26	51.0	0.62	0.43
**Need for TPN**	13	35.1	10	19.6	2.68	0.10
**Need for blood products**	16	43.2	32	62.7	3.29	0.07
**Outcome**					14.10	0.001
Survival to discharge	18	48.6	29	56.9		
Readmission within 30 days	7	18.9	20	39.2		
Death	12	32.4	2	3.9		

* Chi-Square test. MDRI: Multidrug-resistant infections. KAUH: King Abdulaziz University Hospital, IQR: interquartile range, CABSI: catheter-associated blood stream infection, SCA: sickle cell anemia, TPN: total parenteral nutrition.

**Table 4:** Comparison between positive cultures with and without multidrug-resistant bacterial isolates (MDRI) in pediatric patients hospitalized at KAUH between October 2020 and November 2021.

Variables	Cultures with MDRI (N = 223)		Cultures without MDRI (N = 188)		Test *	
	Number	%	Number	%	X^2^	*p*
**Number of cultures/patient (Median: 2, IQR: 1–4)**					0.03	0.86
Single culture/patient	21	56.8	28	54.9		
Multiple cultures/patient	16	43.2	23	45.1		
**Isolated organisms**					46.01	<0.0001
*Klebsiella pneumoniae*	88	39.5	34	18.1		
*Coagulase negative staphylococci (Staphylococcus epidermidis)*	26	11.7	41	21.8		
*Methicillin resistant staphylococcus aureus*	27	12.1	34	18.1		
*Enterobacter coloaca*	5	2.2	3	1.6		
*Escherichia coli*	10	4.5	16	8.5		
*Enterococcus gallinarum*	0	0.0	2	1.1		
*Diphtheroids*	2	0.9	3	1.6		
*Enterococcus faecium*	15	6.7	13	6.9		
*Streptococcus mitis*	2	0.9	1	0.5		
*Salmonella*	0	0.0	3	1.6		
*Serratia marcescens*	0	0.0	7	3.7		
*Pseudomonas aeruginosa*	21	9.4	15	8.0		
*Staphylococcus aureus*	9	4.0	7	3.7		
*Streptococcu viridans*	1	0.4	1	0.5		
*Stenotrophomonas maltophilia*	5	2.2	5	2.7		
*Chryseobacterium meningosepticum*	0	0.0	1	0.5		
*Acinetobacter baumannii*	8	3.6	0	0.0		
*Enterobacter aerogenes*	0	0.0	2	1.1		
**Sites of isolated organisms**					10.78	0.05
Respiratory tract and ETT	80	35.9	46	24.5		
Blood stream and CABSI	83	37.2	93	49.5		
Skin and soft tissue	23	10.3	17	9.0		
Urine/urinary catheter	34	15.2	25	13.3		
Peritoneal fluid	2	0.9	4	2.1		
CSF	1	0.4	3	1.6		

* Chi-Square test. MDRI: Multidrug-resistant infections, KAUH: King Abdulaziz University Hospital, IQR: interquartile range, ETT: endotracheal tube, CABSI: catheter-associated blood stream infection, CSF: cerebrospinal fluid.

**Table 5:** Comparison between deceased patients, surviving patientsand patients who were readmitted to hospital within 30 days.

Variables	Surviving Patients (N = 47)	Readmitted Patients (N = 27)	Deceased Patients (N = 14)	Test *	
				Z	*p*
Age				Z1 = 1.04	p1 = 0.29
(Median and IQR) month	10 (1–24)	36 (7–79)	2 (0.37–54.75)	Z2 = 2.24	p2 = 0.02
Duration of hospital admission				Z1 = 2.39	p1 = 0.02
(Median and IQR) day	22 (14–56)	23 (11–47)	81 (21–191)	Z2 = 2.19	p2 = 0.03
Number of cultures				Z1 = 2.47	p1 = 0.01
(Median and IQR)	2 (1–3)	2 (1–4)	6 (2–14)	Z2 = 1.82	p2 = 0.07
Number of antibiotics used in treatment				Z1 = 2.02	p1 = 0.04
(Median and IQR)	3 (2–5)	3 (2–5)	5 (3–7)	Z2 = 1.87	p2 = 0.06

* Mann–Whitney U test. IQR: Interquartile range, Z1 and p1 for comparing between surviving and deceased patients. Z2 and p2 for comparing between deceased patients and patients readmitted to hospital within 30 days.

## Data Availability

The original contributions presented in the study are included in the article, further inquiries can be directed to the corresponding author.

## References

[1] Moser C., Lerche C.J., Thomsen K., Hartvig T., Schierbeck J., Jensen P.Ø., Ciofu O., Høiby N. (2019). Antibiotic therapy as personalized medicine—General considerations and complicating factors. APMIS.

[2] Gerber J.S., Jackson M.A., Tamma P.D., Zaoutis T.E. (2021). Committee on Infectious Diseases; Pediatric Infectious Diseases Society. Antibiotic Stewardship in Pediatrics. Pediatrics.

[3] Theuretzbacher U. (2012). Accelerating resistance, inadequate antibacterial drug pipelines and international responses. Int. J. Antimicrob. Agents.

[4] Munita J.M., Arias C.A. (2016). Mechanisms of Antibiotic Resistance. Microbiol. Spectr..

[5] Okomo U., Akpalu E.N.K., Le Doare K., Roca A., Cousens S., Jarde A., Sharland M., Kampmann B., Lawn J.E. (2019). Aetiology of invasive bacterial infection and antimicrobial resistance in neonates in sub-Saharan Africa: A systematic review and meta-analysis in line with the STROBE-NI reporting guidelines. Lancet Infect. Dis..

[6] Fleming-Dutra K.E., Hersh A.L., Shapiro D.J., Bartoces M., Enns E.A., File T.M., Finkelstein J.A., Gerber J.S., Hyun D.Y., Linder J.A. (2016). Prevalence of Inappropriate Antibiotic Prescriptions Among US Ambulatory Care Visits, 2010–2011. JAMA.

[7] The Centers for Disease Control and Prevention Antibiotic/Antimicrobial Resistance (AR/AMR): Biggest Threats and Data. www.cdc.gov/DrugResistance/Biggest-Threats.html.

[8] McCullough A.R., Pollack A.J., Plejdrup Hansen M., Glasziou P.P., Looke D.F., Britt H.C., Del Mar C.B. (2017). Antibiotics for acute respiratory infections in general practice: Comparison of prescribing rates with guideline recommendations. Med. J. Aust..

[9] Magiorakos A.P., Srinivasan A., Carey R.B., Carmeli Y., Falagas M.E., Giske C.G., Harbarth S., Hindler J.F., Kahlmeter G., Olsson-Liljequist B. (2012). Multidrug-resistant, extensively drug-resistant and pan drug-resistant bacteria: An international expert proposal for interim standard definitions for acquired resistance. Clin. Microbiol. Infect..

[10] Cosgrove S.E., Carmeli Y. (2003). The impact of antimicrobial resistance on health and economic outcomes. Clin. Infect. Dis..

[11] Jasovský D., Littmann J., Zorzet A., Cars O. (2016). Antimicrobial resistance-a threat to the world’s sustainable development. Ups. J. Med. Sci..

[12] (2014). Review on Antimicrobial Resistance. Tackling a Crisis for the Health and Wealth of Nations. http://amr-review.org/.

[13] Tansarli G.S., Karageorgopoulos D.E., Kapaskelis A., Falagas M.E. (2013). Impact of antimicrobial multidrug resistance on inpatient care cost: An evaluation of the evidence. Expert Rev. Anti-Infect. Ther..

[14] Laxminarayan R., Duse A., Wattal C., Zaidi A.K., Wertheim H.F., Sumpradit N., Vlieghe E., Hara G.L., Gould I.M., Goossens H. (2013). Antibiotic resistance-the need for global solutions. Lancet Infect. Dis..

[15] Ventola C.L. (2015). The antibiotic resistance crisis: Part 1: Causes and threats. Pharm. Ther..

[16] Walther S.M., Erlandsson M., Burman L.G., Cars O., Gill H., Hoffman M. (2002). Antibiotic prescription practices, consumption and bacterial resistance in a cross section of Swedish intensive care units. Acta Anaesthesiol. Scand..

[17] Zhanel G.G., DeCorby M., Laing N., Weshnoweski B., Vashisht R., Tailor F., Nichol K.A., Wierzbowski A., Baudry P.J., Karlowsky J.A. (2008). Antimicrobial-resistant pathogens in intensive care units in Canada: Results of the Canadian National Intensive Care Unit (CAN-ICU) study, 2005–2006. Antimicrob. Agents Chemother..

[18] Al-Faris E.A., Al Taweel A. (1999). Audit of prescribing patterns in Saudi primary health care: What lessons can be learned?. Ann. Saudi Med..

[19] Alenazi F.K., Alzahrani K.M.S., Alhuwaiti M.M., Alshahri A.A. (2022). Antibiotic resistance in Saudi Arabia; review. Int. J. Med. Dev. Ctries..

[20] Shibl A.M., Memish Z.A., Kambal A.M., Ohaly Y.A., Ishaq A., Senok A.C., Livermore D.M. (2014). National surveillance of antimicrobial resistance among Gram-positive bacteria in Saudi Arabia. J. Chemother..

[21] Zowawi H.M., Forde B.M., Alfaresi M., Alzarouni A., Farahat Y., Chong T.M., Yin W.-F., Chan K.-G., Li J., Schrembi M.A. (2015). Stepwise evolution of pandrug-resistance in *Klebsiella pneumoniae*. Sci. Rep..

[22] WHO (2017). Kingdom of Saudi Arabia: National Action Plan on Combating Antimicrobial Resistance. https://www.who.int/publications/m/item/kingdom-of-saudi-arabia-national-action-plan-on-combating-antimicrobial-resistance.

[23] Al-Tawfiq J.A. (2007). Occurrence and antimicrobial resistance pattern of inpatient and outpatient isolates of *Pseudomonas aeruginosa* in a Saudi Arabian hospital: 1998-2003. Int. J. Infect. Dis..

[24] Saeed N.K., Kambal A.M., El-Khizzi N.A. (2010). Antimicrobial-resistant bacteria in a general intensive care unit in Saudi Arabia. Saudi Med. J..

[25] Almaghrabi M.K., Joseph M.R.P., Assiry M.M., Hamid M.E. (2018). Multidrug-Resistant *Acinetobacter baumannii*: An Emerging Health Threat in Aseer Region, Kingdom of Saudi Arabia. Can. J. Infect. Dis. Med. Microbiol..

[26] Halwani M.A., Tashkandy N.A.J., Aly M.M., Al Masoudi S.B., Dhafar O.O. (2015). Incidence of Antibiotic Resistance Bacteria in Jeddah’s Ministry of Health Hospitals, Saudi Arabia. Adv. Microbiol..

[27] Alam M.Z., Alam Q., Fatani A.H.J., Shukri H.A., Haque A. (2017). A surveillance study on the prevalence and antimicrobial resistance pattern among different groups of bacteria isolated from Western province of Saudi Arabia. Biomed. Res..

[28] Chen Y., Chen X., Liang Z., Fan S., Gao X., Jia H., Li B., Shi L., Zhai A., Wu C. (2022). Epidemiology and prediction of multidrug-resistant bacteria based on hospital level. J. Glob. Antimicrob. Resist..

[29] Chen X., Pan D., Chen Y. (2021). The drug resistance of multidrug-resistant bacterial organisms in pediatric pneumonia patients. Am. J. Transl. Res..

[30] Rizwan M., Fatima N., Alvi A. (2014). Epidemiology and pattern of antibiotic resistance in *Helicobacter pylori*: Scenario from Saudi Arabia. Saudi J. Gastroenterol..

[31] Khan M.A., Faiz A. (2016). Antimicrobial resistance patterns of *Pseudomonas aeruginosa* in tertiary care hospitals of Makkah and Jeddah. Ann. Saudi Med..

[32] Balkhy H.H., Memish Z.A., Almuneef M.A., Cunningham G.C., Francis C., Fong K.C., Nazeer Z.B., Tannous E. (2007). Methicillin-resistant *Staphylococcus aureus*: A 5-year review of urveillance data in a tertiary care hospital in Saudi Arabia. Infect. Control Hosp. Epidemiol..

[33] Alshammari F., Cruz J.P., Alquwez N., Almazan J., Alsolami F., Tork H., Alabdulaziz H., Felemban E.M. (2018). Compliance with standard precautions during clinical training of nursing students in Saudi Arabia: A multi-university study. J. Infect. Dev. Ctries..

[34] Zhang J., Yu W., Zhao L., Xiao Y. (2016). Epidemiology and characteristics of antibacterial resistance in China. Chin. J. Clin. Infect. Dis..

[35] Agyepong N., Govinden U., Owusu-Ofori A., Essack S.Y. (2018). Multidrug-resistant gram-negative bacterial infections in a teaching hospital in Ghana. Antimicrob. Resist. Infect. Control.

[36] Sritippayawan S., Sri-Singh K., Prapphal N., Samransamruajkit R., Deerojanawong J. (2009). Multidrug-resistant hospital-associated infections in a pediatric intensive care unit: A cross-sectional survey in a Thai university hospital. Int. J. Infect. Dis..

[37] Rezk A.R., Bawady S.A., Omar N.N. (2021). Incidence of emerging multidrug-resistant organisms and its impact on the outcome in the pediatric intensive care. Egypt Pediatr. Assoc. Gaz..

[38] Chandra R.K. (1999). Nutrition and immunology: From the clinic to cellular biology and back again. Proc. Nutr. Soc..

[39] González-Torres C., González-Martínez H., Miliar A., Nájera O., Graniel J., Firo V., Alvarez C., Bonilla E., Rodríguez L. (2013). Effect of malnutrition on the expression of cytokines involved in Th1 cell differentiation. Nutrients.

[40] Ahmed M., Mirambo M.M., Mushi M.F., Hokororo A., Mshana S.E. (2017). Bacteremia caused by multidrug-resistant bacteria among hospitalized malnourished children in Mwanza, Tanzania: A cross sectional study. BMC Res. Notes.

[41] Holowka T., van Duin D., Bartelt L.A. (2023). Impact of childhood malnutrition and intestinal microbiota on MDR infections. JAC Antimicrob. Resist..

[42] Walson J.L., Berkley J.A. (2018). The impact of malnutrition on childhood infections. Curr. Opin. Infect. Dis..

[43] Wang Z., Xia Z. (2020). What we can do? The risk factors for multi-drug resistant infection in pediatric intensive care unit (PICU): A case-control study. Ital. J. Pediatr..

